# Monitoring neurodegeneration in diabetes using adult neural stem cells derived from the olfactory bulb

**DOI:** 10.1186/scrt201

**Published:** 2013-05-14

**Authors:** Ryo Hidaka, Masanao Machida, Shin Fujimaki, Kazuyuki Terashima, Makoto Asashima, Tomoko Kuwabara

**Affiliations:** 1 Research Center for Stem Cell Engineering, National Institute of Advanced Industrial Science and Technology (AIST), Central 4, 1-1-4 Higashi, Tsukuba Science City 305-8562, Japan

**Keywords:** Neural stem cells, Hippocampus, Olfactory bulb, Diabetes, Insulin

## Abstract

**Introduction:**

Neurons have the intrinsic capacity to produce insulin, similar to pancreatic cells. Adult neural stem cells (NSCs), which give rise to functional neurons, can be established and cultured not only by intracerebral collection, which requires difficult surgery, but also by collection from the olfactory bulb (OB), which is relatively easy. Adult neurogenesis in the hippocampus (HPC) is significantly decreased in diabetes patients. As a result, learning and memory functions, for which the HPC is responsible, decrease.

**Methods:**

In the present study, we compared the effect of diabetes on neurogenesis and insulin expression in adult NSCs. Adult NSCs were derived from the HPC or OB of streptozotocin-induced diabetic rats. Comparative gene-expression analyses were carried out by using extracted tissues and established adult NSC cultures from the HPC or OB in diabetic rats.

**Results:**

Diabetes progression influenced important genes that were required for insulin expression in both OB- and HPC-derived cells. Additionally, we found that the expression levels of several genes, such as voltage-gated sodium channels, glutamate transporters, and glutamate receptors, were significantly different in OB and HPC cells collected from diabetic rats.

**Conclusions:**

By using identified diabetes-response genes, OB NSCs from diabetes patients can be used during diabetes progression to monitor processes that cause neurodegeneration in the central nervous system (CNS). Because hippocampal NSCs and OB NSCs exhibited similar gene-expression profiles during diabetes progression, OB NSCs, which are more easily collected and established than HPC NSCs, may potentially be used for screening of effective drugs for neurodegenerative disorders that cause malignant damage to CNS functions.

## Introduction

Adult neuronal stem cells (NSCs) exist in the hippocampus (HPC), which is responsible for learning and memory, and new neurons are continuously generated, even in the adult brain [1-3]. Adult NSCs retain the self-renewal ability characteristic of stem cells and can give rise to three differentiation lineages: neurons, astrocytes, and oligodendrocytes [4,5]. As shown in recent studies, the typical neurogenic regions in the mammalian brain are the HPC, the subventricular zone (SVZ), and the olfactory bulb (OB), and adult NSC cultures can be established from these regions. NSCs derived from the OB have the potential to be used in therapeutic applications because they are easily harvested without harm to the patient [6,7].

The fate of multipotent NSCs is determined by diverse extracellular signals, corresponding intrinsic transcriptional factors, and epigenetic machineries. Insulin is an important neuromodulator, contributing to neurobiologic processes, energy homeostasis, and cognitive function. Moreover, insulin increases the tolerance of mature neurons to toxicity and has a protective function that keeps the network functions of the neurons in an active state [8,9]. Insulin plays a major role in controlling the differentiation fate of NSCs. Insulin also promotes the induction of undifferentiated NSCs to differentiate into oligodendrocytes, which function in protecting neurons [10]. Moreover, insulin promotes the function of fibroblast growth factor 2 (FGF-2), which has an important role in maintaining NSCs in the undifferentiated state, and also plays a major role in the stem cell self-renewal stage (that is, it strongly activates stem cell proliferation [1,11-14]).

In the presence of low levels of insulin, the proliferative functions of undifferentiated stem cells are suppressed in many organs. Insulin-mediated signal transduction regulates multiple roles in the self-renewal and differentiation pathways of adult stem cells. Diabetes impairs the function of hematopoietic stem cells [15], skeletal muscle stem cells (satellite cells) [16,17], osteoblast stem cells [18], and NSCs [19,20]. The diabetes-induced reduction in adult neurogenesis has been detected mainly in the HPC, and diabetes has been associated with a decline in the cognitive functions of learning and memory. Not only are diabetes patients at increased risk of contracting neurodegenerative diseases and psychiatric disorders, such as Alzheimer disease, Parkinson disease, depression, and Huntington disease [19,21,22], but diabetes has also been experimentally shown to have a great effect on the functions of the neural circuits in the HPC [8,23,24]. Streptozotocin (STZ)-induced diabetes produces a dramatic decrease in cell proliferation in the hippocampal dentate gyrus (DG) of rodents, and a significant decrease in the number of BrdU(+) cells has been detected 2 days after STZ-induction [25], suggesting that the effects of decreasing insulin-mediated NSC regulation during diabetes progression are acute. Reductions in NSC proliferation have been observed not only in the HPC but also in the SVZ [26].

Although recent studies indicate that anosmia is associated with diabetes [27] and that insulin can modulate olfactory processing for the OB network [28], the effect of diabetes (that is, the decline in insulin regulation) on OB NSC fate determination and potential genes involved in the insulin-dependent regulation of OB NSCs are unknown.

We recently demonstrated that adult OB and HPC NSCs collected from diabetic rats are useful cell sources for autologous cell transplantation to treat diabetes because they retain the intrinsic ability to produce insulin in the adult CNS [29]. In this study, we examined the changes in gene expression required for neuronal differentiation and insulin expression in adult OB NSCs during diabetes progression. The properties of OB NSCs in response to diabetes pathogenesis were compared with those of HPC NSCs. Identification of genes that are similarly altered in the HPC and OB during the progression of diabetes would be useful to monitor and/or to investigate the functions of cells in the HPC during diabetes progression in the clinical setting, because OB NSCs can be easily collected and cultured from patients.

## Materials and methods

### Animals

Experiments were carried out on 30 Fischer 344 male rats (about 4 months old) weighing 120 to 160 g. Diabetes mellitus was induced in 14 animals by single intraperitoneal injection of 50 mg/kg streptozotocin (Wako, Osaka, Japan) dissolved in 0.5 ml of citrate buffer (0.1 *M*, pH 4.5). The blood glucose levels were measured every 1 to 2 days, and rats with blood glucose levels greater than 300 mg/dl were considered diabetic [29]. Rats of the experimental group were injected with streptozotocin, and animals of the control group (*n* = 7) were injected with an analogous volume of citrate buffer.

Tissue samples for expression-profile studies were collected at 5 days, 2 weeks, and 2 months of streptozotocin-induced diabetes. Animals were anesthetized with pentobarbital sodium (70 to 100 mg/kg), and then transcardially perfused sequentially with phosphate buffer (0.1 *M*) and 4% PFA solution. All animal procedures were performed according to a protocol approved by the Institutional Animal Care and Use Committee (IACUC) of the National Institute of Advanced Industrial Science and Technology.

### Cell preparation and the culture

Male Fisher 344 rats with a body weight of 100 to 150 g 10 days after the streptozotocin induction were used (Charles River Japan, Inc, Yokohama, Japan). Adult hippocampal NSCs were prepared and maintained as described previously [2,29]. Rats were anesthetized, and the head was fixed in a stereotactic frame. The olfactory bulb (OB) (AP4.2; L1.0; U2.5) and hippocampal dentate gyrus (DG) (AP -3.6; -L2.8; U3.0) were taken. Tissue samples were transferred to ice-cold PBS solution (Wako). Collected HPC or OB was microdissected and dissociated by digestion with a mixture of papain (Worthington Biochemical Corporation, NJ, USA), dispase (Worthington Biochemical Corporation, NJ, USA), deoxyribonuclease (Worthington), and StemPro Accutase (Invitrogen). The cell mixture was passed through a 40-μm cell strainer (BD Falcon, Tokyo, Japan) to obtain a single-cell suspension. The resulting cell suspension was washed with Dulbecco modified Eagle medium/F-12 medium (DMEM/F12; Invitrogen, Life Technologies Japan Ltd., Tokyo, Japan), including antibiotic-antimycotic and FGF2, several times after centrifugation. Purified HPC or OB cells were incubated in DMEM/F12 containing 100 ng/ml FGF-2 and N2 Supplement with Transferrin (Apo, Wako) on poly-ornithine-laminin-coated dishes that had been determined to be suitable for culturing both OB NSCs and HPC NSCs [29,30]. The media contained elevated levels of FGF2 (100 ng/ml) during the initial culture before the first passage with StemPro Accutase. Stably proliferating NSCs were cultured with DMEM/F12 containing 20 ng/ml FGF2, 1% N2 supplement, 1% antibiotic-antimycotic, and 2 m*M* L-glutamine in a 5% CO_2_ incubator at 37°C.

We cultured adult HPC and OB NSCs simultaneously under FGF2 in all experiments performed in this parallel characterization study. For neuronal differentiation, cells were cultured in DMEM/F12 medium containing retinoic acid (RA) (1 μ*M*, Sigma-Aldrich Japan K.K., Tokyo, Japan), forskolin (5 μ*M*, Sigma-Aldrich Japan K.K., Tokyo, Japan) and KCl (40 m*M*, Wako).

### Western blotting and immunoprecipitation

The sample was homogenized in lysis buffer (50 m*M* Hepes, pH 7.4, 150 m*M* NaCl, 2 m*M* EDTA, 1% sodium deoxycholate, 1% NP-40, 0.2% sodium dodecylsulfate) containing a phosphatase inhibitor and protease inhibitor mix (Nakarai Tesque Inc., Kyoto, Japan) on ice. Protein concentrations were measured by using a BCA protein-assay kit (Thermo Fisher Scientific K.K., Kanagawa, Japan). Each homogenized sample was diluted with SDS-PAGE loading buffer (62.5 m*M* Tris–HCl, pH 6.8, 2% wt/vol SDS, 10% glycerol, 50 m*M* β-mercaptoethanol, 0.01% wt/vol bromophenol blue) to 2.0 mg/ml, and an equivalent volume of each sample was loaded onto 5% to 20% polyacrylamide gel (Wako Pure Chemical Industries, Ltd., Osaka, Japan). An electrically blotted PVDF membrane (Nihon Millipore, Tokyo, Japan) was subjected to blocking with Blocking One (Nakarai Tesque Inc.) for 1 hour at room temperature. Antibodies against SCN1B (Abcam, Tokyo, Japan) and Neurexin I (BD Japan, Tokyo, Japan) were diluted 1:5,000 with TBS containing 0.05% Tween 20 (TBST) and used as the primary antibodies, and incubation was performed for 12 hours at 4°C. Anti-rabbit or anti-mouse IgG-conjugated HRP (GE Healthcare Japan, Tokyo, Japan), diluted 1:50,000 with blocking buffer, was used as the secondary antibody, and incubation was performed for 1 hour at room temperature. After incubation with SuperSignal West Femto Maximum Sensitivity Substrate (Thermo Scientific Japan, Yokohama, Japan), the result was imaged by using the LAS-3000 Imaging system (Fuji Film Corporation, Tokyo, Japan). After a careful wash with blocking buffer, SuperSignal West Femto Maximum Sensitivity Substrate (Thermo Scientific Japan, Yokohama, Japan) was used as the chromogen. The bands on the PVDF membrane were analyzed by using NIH Image J.

For immunoprecipitation (IP) analysis, collected tissue-derived lysates were washed twice with PBS and then resuspended in IP-lysis buffer (50 m*M* HEPES/KOH, pH 7.5, 50 m*M* potassium acetate, 8 m*M* MgCl_2_, 2 m*M* EGTA, and 50 μg/ml digitonin) on ice for 10 minutes. To prepare Protein G agarose (Millipore), the beads were washed twice with PBS and restored to a 50% slurry with PBS. The lysate was pre-cleared by adding 100 μl of G agarose bead slurry (50%) per 1.5 ml of lysate and incubating at 4°C for 10 minutes on an orbital shaker, and the Protein G beads were removed by centrifugation at 14,000 *g* at 4°C for 10 minutes. The supernatant was transferred to a fresh centrifuge tube as the pre-cleaned fraction. The fractions were incubated overnight at 4°C with 20 μl of normal rabbit serum in binding buffer (20 m*M* Tris–HCl, pH 7.5, 60 m*M* KCl, 2.5 m*M* EDTA, and 0.1% Triton X-100) and suspended with protein G-agarose beads. After removal of the nonspecific binding fraction to beads by precipitation, the resulting supernatant lysate was mixed with 100 μl of protein G-agarose beads plus 1 to 5 μl (corresponding to 1 μg) of each specific antibody. After incubation at 4°C overnight, the beads were washed 5 times with lysis buffer (50 m*M* Hepes-KOH, pH 7.5, 60 m*M* KCl, 2.5 m*M* EDTA, and 0.1% Triton X-100). Proteins were eluted by boiling the beads and were separated with SDS-PAGE for after Western blotting detection.

### RNA extraction and qPCR analysis

Total cellular RNA was isolated by using the ISOGEN (Wako). RNA from dissected DG of HPC was purified with ISOGEN after homogenization (Microson, Heat Systems). Total RNA was treated RNase-free DNase I (Ambion, Life Technologies Japan Ltd., Tokyo, Japan). First-strand cDNA synthesis was carried out by following the manufacturer’s protocol (Invitrogen, Life Technologies Japan Ltd., Tokyo, Japan; Takara Bio Inc., Shiga, Japan). Quantitative PCR was performed by using the SyBr Green method (Applied Biosystems; 4309155) and standard 35 to 40 cycles by using the ABI qPCR machine. GAPDH was used as an internal control.

The ΔΔCT method of relative quantification was used to determine the fold change in gene expression. The ΔΔCT calculation was done by first normalizing the resulting threshold cycle (CT) values of the target mRNAs to the CT values of the internal control GAPDH in the same samples (ΔCT = CT_Target_ - CT_GAPDH_ ). It was further normalized with the control (ΔΔCT = ΔCT - CT _Control_). The fold change in expression was then obtained (2^-ΔΔCT^).

### Statistics

All experiments were analyzed for statistical significance by using a Student *t* test, with all error bars expressed as ± standard error of the mean (SEM). Values of *P* < 0.05 were considered significant.

## Results and discussion

### Effects of diabetes on OB and HPC tissues: time-course analysis of gene expression required for insulin expression and neuronal differentiation after induction of diabetes by STZ

Intraperitoneal injection of STZ caused the development of pronounced hyperglycemia in experimental animal [29]; the blood glucose levels of STZ-induced rats were greater than 300 mg/dl on the third day after the injection. On day 5 of STZ-induced hyperglycemia, the brains were removed quickly, and the DG region was microdissected to extract total RNA. For qPCR analysis, RNAs from the DGs of control and diabetic rats were extracted and were subjected to qPCR to analyze the expression of *Sox2*, *Nestin*, *NeuroD1*, *insulin*, *beta*-*tubulin III* (*TUBB3*), *synapsin 1* (*SYN1*), *glial fibrillary acidic protein* (*GFAP*), *GLIT1*, *SC1*, and *bystin*-*like* (*BYSL*, astrocyte marker genes) [31]. RNA samples were also prepared from rats 2 weeks and 2 months after STZ induction to observe changes in the gene-expression profiles, depending on the time course of STZ-induced diabetes.

**Figure 1:**
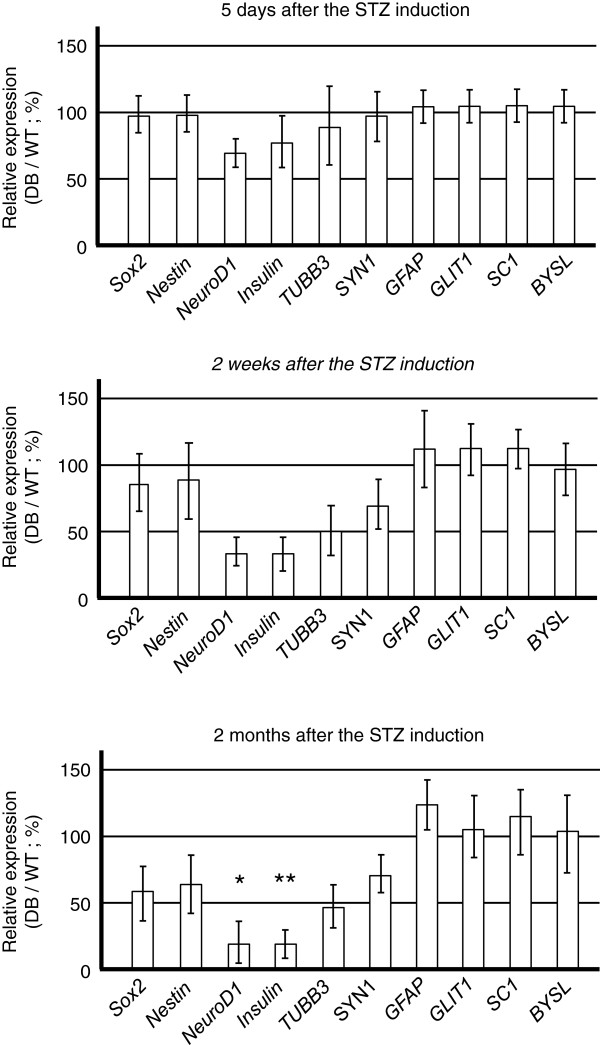
**Changes in the DG expressions of genes controlling adult HPC neurogenesis during diabetes progression.** On the day 5 of streptozotocin (STZ)-induced hyperglycemia, the brains were removed quickly, and the dentate gyrus (DG) region was microdissected to extract total RNA. For qPCR analysis, RNAs from the DG of control (*n *= 6) and diabetic rats (*n *= 6) were extracted and were subjected to qPCR analysis of gene expression of *Sox2 *and *Nestin *(stem cell marker genes), *NeuroD1 *(insulin-activating gene), *insulin *(proinsulin1), *β-tubulin III *(TUBB3, immature neuron marker gene), *SYN1 *(mature neuron marker gene), *GFAP*, *GLIT1*, *SC1*, and *BYSL* (astrocyte-marker genes). RNA samples were also prepared from rats 2 weeks and 2 months after the STZ induction.

**Figure 2:**
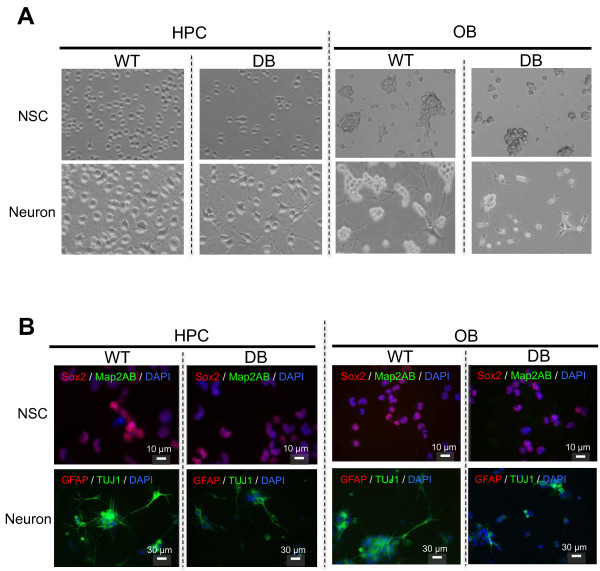
**Differentiation abilities of adult NSCs derived from HPC and OB of diabetes rats.** (**A**) Adult NSCs cultures derived from HPC and OB of diabetes rats. The adult NSCs isolated from HPC of control wild-type rats (WT) and diabetes (DB) rats are shown in left panels. The adult NSCs isolated from OB are shown in right panels. *In vitro* differentiated adult HPC- and OB neurons are also shown (Neuron). When the culture was exposed to neuron-differentiation conditions (RA + FSK + KCl), both HPC and OB cells extended neuritis. (**B**) Immunohistochemistory analysis (IHC) of the adult NSCs isolated from HPC (left) and OB (right) of control wild-type rats (WT) and diabetes (DB). IHC images using antibodies of Sox2 (red), Map2AB (green) are shown in the upper panels. IHC images using antibodies of GFAP (red), TUJ1 (green) were shown in lower panels.

Expression of *Sox2* and *Nestin* mRNA was similar in control rats (WT) and STZ-induced diabetic rats (DB) on day 5 (top panel in Figure 1; relative expression in DB samples/WT sample (%)). Additionally, the expression of astrocytic genes was unchanged (*GFAP*, *GLIT1*, *SC1,* and *BYSL*). However, reduced expression of *NeuroD1* and *insulin* transcripts in the hippocampal DG was observed during the early progression of diabetes (5 days after STZ induction; Figure 1, top panel). The reduced expression of these targets was maintained at both the 2-week (middle panel, Figure 1) and 2-month time points (bottom panel, Figure 1), indicating that diabetes progression was associated with a decline in insulin expression in the hippocampal DG.

After the significant reduction of *NeuroD1* and *insulin* mRNA expression in the DG of diabetic rats (*P = 0.01; **P = 0.001; Figure 1), the expression of neuronal genes such as *TUBB3* and *SYN1* was also decreased. However, the expression of astrocytic genes remained unchanged, suggesting that diabetes caused inhibitory effects on the differentiation of NSCs into neuronal lineages. Because NeuroD1 is indispensable for triggering neuronal differentiation in adult hippocampal NSCs [32,33], the decline in NeuroD1 expression may directly affect neuronal differentiation and influence mature neuronal genes, such as *SYN1*. Additionally, because NeuroD1 is necessary for insulin expression in adult NSCs via direct transcriptional activation of the bHLH transcription factor [29], a reduction in insulin expression would be expected to occur simultaneously. Moreover, insulin is known to promote the function of FGF-2, indicating that insulin also enhances and supports the self-renewal ability of stem cells. Therefore, the reduction in insulin expression in diabetes at early time points may indirectly inhibit the function of the stem cell population.

The expression of *Sox2* and *Nestin* transcripts (stem cell marker genes) was reduced at the 2-month time point (bottom panel, Figure 1), suggesting that diabetes caused impairment of adult neurogenesis, consistent with previous study [24]. Diabetes-mediated inhibition of stem cell abilities in the HPC is probably associated with changes in the expression of the *Sox2* gene, because Sox2 protein is critical for maintaining NSC function (both proliferation and self-renewal abilities) [5,34-36]. Moreover, the involvement of Sox2 in the regulation of NeuroD1 via the Sox/LEF regulatory system and Sox2-mediated suppression of genes is required for differentiation in undifferentiated NSCs and for Wnt (TCF/LEF)-mediated activation of such genes [32].

### Effects of diabetes on OB and HPC NSCs cultures: the establishment of adult NSCs from the OB and HPC of rats during the early phase of diabetes progression

The predominant effects of diabetes progression on neuronal differentiation were observed *in vivo* with qPCR analysis by using hippocampal DG samples collected at early time points (that is, 5 days to 2 weeks after STZ induction; Figure 1). We established adult NSC cultures from hippocampal DG cells of control rats (WT, wild type) and STZ-induced diabetic rats (DB) at 10 days after induction. To investigate the utility of OB-derived adult NSCs for monitoring potential changes that may reflect diabetes-mediated neurodegeneration in the CNS, we established adult NSC cultures from the OB of WT and diabetic rats at the same time point (10 days after the induction).

In parallel, adult HPC- and OB-derived NSC cultures were maintained in 20 ng/ml FGF-2 (Figure 2). Adult NSC cultures established from the hippocampal DG from WT or diabetic animals exhibited similar morphologies (NSC; Figure 2A). HPC NSCs from both groups of rats were round and retained their shapes when expanded as a monolayer. HPC and OB NSCs derived from WT rats expressed *Sox2* transcripts, but Sox2 expression was more obvious in NSCs derived from WT rats than in those derived from diabetic rats (Figure 2B). Because immunohistochemistry (IHC) analysis showed that Sox2(+) HPC NSCs did not express Map2ab, NSCs treated with the FGF-2 ligand did not enter neuronal differentiation (Figure 2B).

Under neuronal differentiation conditions (Neuron), the cell morphologies changed markedly, and extended neurites were observed (Figure 2A). In HPC neuron cultures from WT animals (WT HPC neuron), multiple neurites were generated from a cell body, and they were extended in various directions in a complex manner. In contrast, in HPC neurons derived from diabetic animals (DB HPC neuron), fewer neurite connections were created than those observed in WT HPC neurons (Figure 2A). The differentiation properties of WT HPC neurons and diabetic HPC neurons were also evaluated by using IHC analysis. In the NSC state, NSCs from both the WT HPC and the diabetic HPC exhibited positive signals for Sox2 in their nuclei, whereas Map2ab staining was negative (Figure 2B), indicating that these NSCs were retained in the undifferentiated stem cell state. In contrast, after neuronal differentiation, neurons from both the WT HPC and the diabetic HPC expressed TUJ1, but not GFAP, indicating that these cells had successfully committed to neuronal differentiation lineages. Notably, the intensity of the TUJ1-positive signal was stronger in neurons from the WT HPC than in neurons from the diabetic HPC (Figure 2B).

OB NSCs from WT or diabetic rats grew as heterogeneous cultures with adherent properties and neurosphere morphologies (Figure 2A). In OB NSCs from diabetic animals (DB OB NSC), neurospheres formed with inconsistent shapes and sizes, and dying cells (single cells) were more frequently observed than in OB NSC cultures from control animals (WT DB NSC). The adherent properties of OB NSCs from diabetic rats seemed weaker than those of OB NSCs from WT rats (Figure 2A). In OB neuron cultures from WT animals, multiple and prolonged neurites were generated from clusters of cells (WT OB neuron, Figure 2A). WT OB neurons differentiated from WT OB NSCs *in vitro* exhibited extended neurites that were positive for TUJ1 in IHC analysis (Figure 2B). In contrast, OB neurons differentiated from diabetic OB NSCs (DB OB neurons) created thinner and shorter neurites from the cell body than those observed in WT OB neurons (Figure 2B). IHC analysis of OB neurons derived from diabetic rats indicated that these cells had difficulties with neuronal differentiation because few TUJ1-positive neurons were found in the culture (Figure 2B).

These results suggest that diabetes caused inhibitory effects on the neurogenesis of adult NSCs in both *in vivo* and *in vitro* cultures derived from the HPC and OB of diabetic animals. Adult NSC cultures were established at early period during the progression of diabetes (10 days after the STZ induction), and this early time point was sufficient for the detection of differences in the function of adult NSCs between WT and diabetic rats. Differences became clearer when NSCs derived from diabetic rats were differentiated into neuronal lineages, both in the HPC and DB (Figure 2).

### Identification of diabetes-response genes in adult OB and HPC NSCs derived from the early phase of diabetes progression: Wnt signaling-related molecules

**Figure 3:**
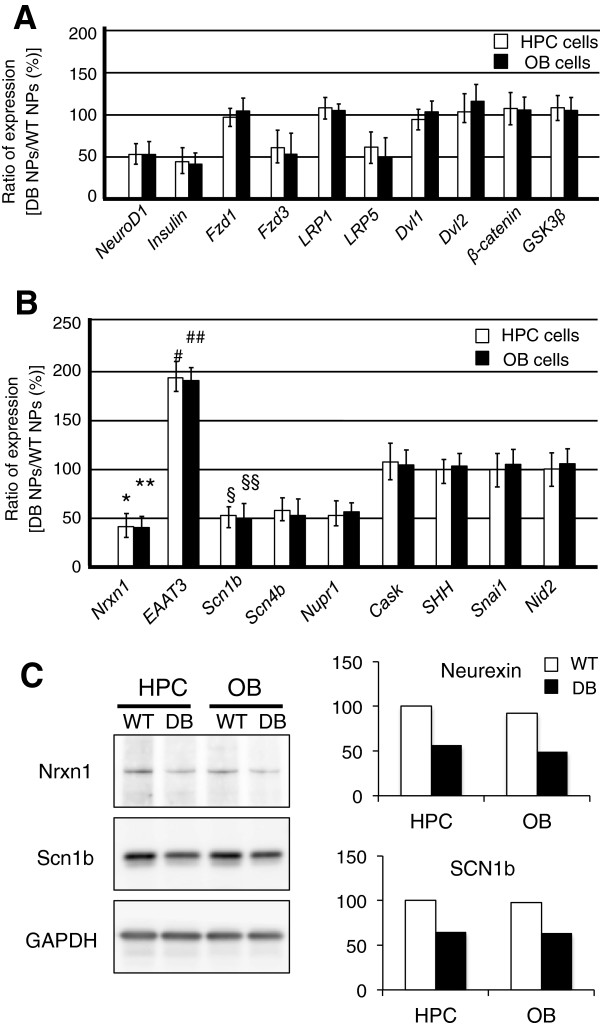
**Identification of diabetes-response genes in adult OB and HPC neurons.** (**A**) qPCR analysis of diabetes-response genes related to Wnt signaling. WT (wild-type) HPC NSCs, DB (diabetic) HPC NCSs, WT OB NSCs, and DB OB NSCs were differentiated simultaneously into neuronal lineages, and the total RNAs were extracted 24 hours after the neuronal induction (neuronal progenitor stage; NP). (**B**) qPCR analysis of diabetes-response genes in OB and HPC neurons. (**C**) Western blotting analysis of diabetes-response genes. *Nrxn1 *(neurexin 1) exhibited DB-specific downregulation (top). Similarly, *Scn1b *(sodium channel, voltage-gated, type I, β subunit) shows diabetes-associated reduction (middle). Relative band intensity of each protein in the Western blotting analysis is shown on the right.

To determine specific genes that may account for the differences in the neuronal differentiation of diabetic NSCs and WT NSCs, we next conducted comparative qPCR analysis. NSCs derived from the WT and diabetic HPC and OB were differentiated simultaneously into neuronal lineages, and total RNA was extracted 24 hours after the neuronal induction (NP, neuronal progenitor stage). Our previous microarray study indicated the general gene profiles that are up- or downregulated on neuronal induction in adult NSCs (and subsequently formed neurons) [29]. Genes responding similarly in NSCs from both the HPC and OB at early phases of diabetes progression have the potential to be used as marker genes in monitoring disease-associated alterations of HPC cells by using OB NSC cultures (Figure 3).

After neuronal differentiation, as described earlier, the expression of *NeuroD1* and *insulin* transcripts was reduced specifically in OB and HPC NSC cultures derived from diabetic rats (Figure 3A). Because the activation of *NeuroD1* mRNA expression requires Wnt signaling [29,32], we examined the intracellular expression of Wnt-related molecules in our analysis. The frizzled (Fzd) receptor is associated with a transmembrane protein called low-density lipoprotein receptor-related protein (LRP) in Wnt signal transduction. Wnt signaling through Fzd and LRP receptor pairs activates dishevelled (Dvl) and inhibits glycogen synthase kinase (GSK) 3β, and then stabilizes β-catenin. The expression of *Fzd1*, *LRP1*, *Dvl1*, *Dvl2*, *β*-*catenin*, and *GSK3β* mRNAs was almost unchanged between diabetic and WT samples in both OB and HPC NSC cultures under the neuronal differentiation conditions used in this study (Figure 3A).

From qPCR analysis, we found that the expression of *Fzd3* and *LRP5* mRNAs was coordinately downregulated in both OB and HPC cultures, specifically in samples derived from diabetic rats. Fzd3 is a transmembrane receptor for secreted Wnt glycoproteins involved in the Wnt signal-transduction cascade. Association studies have shown that the *Fzd3* gene plays an important role in underlying schizophrenia [37]. Our data suggest that Fzd3 may be involved in Wnt signaling pathways that influence the expression of NeuroD1 [29,32] in adult NSCs during the progression of diabetes.

LRP5 is a novel member of the LRP receptor family in the Wnt-signaling cascade and is genetically associated with type 1 diabetes [38]. Polymorphisms in the promoter region of LRP5 are associated with diabetes [38], and alterations in LRP5 expression may be responsible for diabetes susceptibility [38,39]. The reduction in LRP5 expression in both OB and HPC NSC cultures derived from diabetic animals implied that LRP5 may influence the neurodegenerative phenotype in the CNS, especially in the HPC and OB under diabetic conditions.

### Diabetes-response genes in adult OB and HPC NSCs during neuronal differentiation: potential marker genes to detect the malignancy of CNS function by using adult NSCs derived from the OB

Among many neuronal lineage-specific genes expressed in hippocampal neurons, most are equally expressed in OB neurons. Cask (calcium/calmodulin-dependent serine protein kinase), SSH (sonic hedgehog), Snai1 (snail homolog 1, zinc finger protein), and Nid2 (nidogen 2; basement membrane proteins) were highly expressed in both OB and HPC neurons *in vitro*, consistent with microarray analysis of *in vitro* cultures of OB and HPC neurons [29], and the expression levels of these targets were similar between diabetic and WT samples (Figure 3B).

From qPCR analysis, we identified five diabetes-response genes in adult NSCs that were similarly altered (that is, either increased or decreased) during neuronal differentiation in OB and HPC *in vitro* cultures. *Nrxn1* (neurexin 1), *Scn1b* (sodium channel, voltage-gated, type I, β subunit), *Scn4b* (sodium channel, voltage-gated, type IV, β subunit) and *Nupr1* (nuclear protein 1) transcripts were downregulated only in OB and HPC neurons derived from diabetic rats. Nrxn1 belongs to a neurexin family of proteins that function in the vertebrate nervous system as cell-adhesion molecules and receptors. Mutation of the *Nrxn1* gene has been associated with schizophrenia, autism, intellectual disability, and type 1 diabetes [40]. Western blotting analysis with specific antibodies against *Nrxn1* and *Scn1b* revealed that the observed diabetes-associated reduction in *Nrxn1* and *Scn1b* mRNA expression in HPC and OB neurons was also seen at the protein level (Figure 3C).

Overexpression of the *Nupr1* gene has been shown to enhance glucose-stimulated β-cell proliferation and insulin secretion in primary human islets [40-43], indicating that the primary role of the *Nupr1* gene is the regulation of insulin in pancreatic endocrine cells. The adult CNS (neurons) and endocrine system (β cells) share common transcription factors, such as NeuroD1, that are required for insulin expression [29]. They also use similar cellular signaling pathways (that is, Wnt signaling is required for the activation of the *NeuroD1* gene) via the secretion of Wnt3 from their niches (astrocytes and α cells) [29,44]. Diabetes-specific downregulation of *Nupr1* transcripts in adult OB and HPC neurons at early phases of diabetes progression suggest that Nupr1 affects and accelerates the dysregulation of insulin-mediated intra- and intercellular networks in the CNS, promoting the pathogenesis of neurodegenerative disorders.

**Figure 4:**
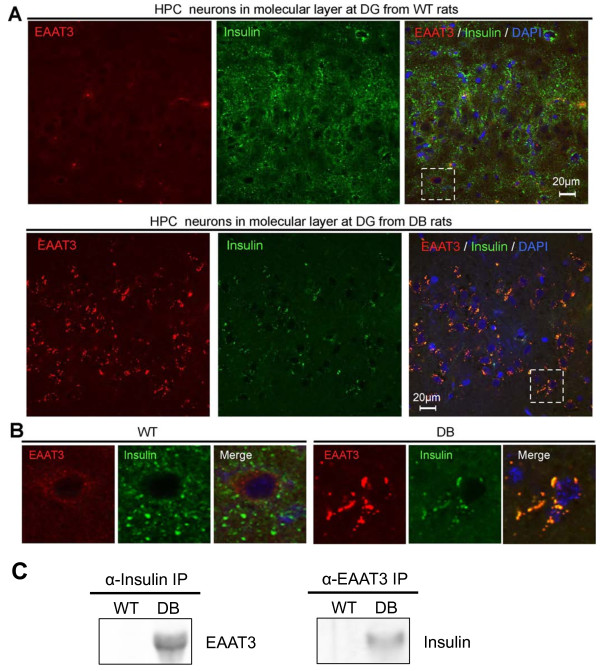
**Upregulated EAAT3 interacts with insulin under diabetes in HPC.** (**A**) IHC of HPC neurons in molecular layer at DG of WT (wild-type) and DB rats. IHC images using antibodies of EAAT3 (red) and Insulin (green) are shown. (**B**) Magnified image at dotted-line area in Figure 4A is shown in separate panels. (**C**) Immunoprecipitation (IP) analysis of EAAT3 and insulin. The association between EAAT3 and insulin was specifically observed in the HPC from diabetic rats when EAAT3 expression was highly upregulated.

**Figure 5:**
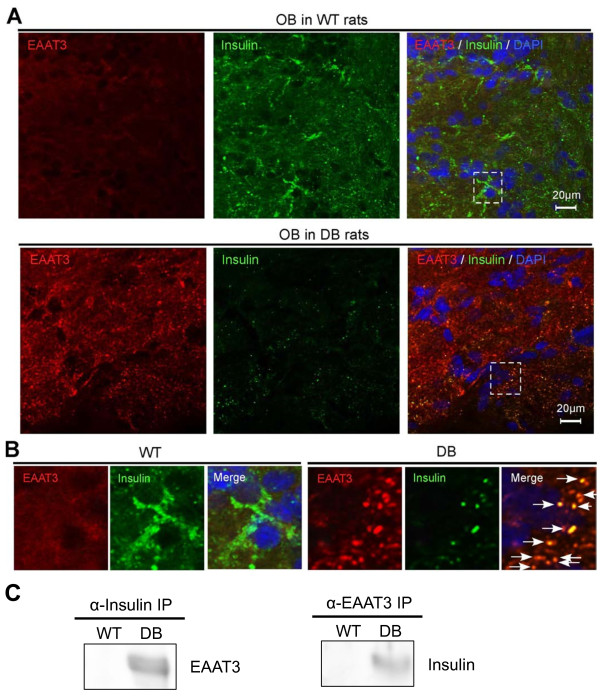
**Upregulated EAAT3 interacts with insulin in diabetes in OB.** (**A**) IHC analysis of EAAT3 and Insulin in OB. IHC images using antibodies of EAAT3 (red) and Insulin (green) are shown. (**B**) Magnified image at dotted line area in Figure 5A is shown in separate panels. (**C**) IP analysis of EAAT3 and insulin in OB. The association between EAAT3 and insulin was specifically observed in the OB from diabetic rats when EAAT3 expression was highly upregulated.

In contrast, excitatory amino acid transporters (EAATs), also known as glutamate transporters, were highly upregulated specifically in OB and HPC neurons derived from diabetic NSCs (Figure 3B). Our subsequent IHC analysis of EAAT3 in the HPC (Figure 4) and OB (Figure 5) revealed colocalization of EAAT3 and insulin proteins specifically in diabetic animals. Compared with WT rats (Figure 4A, upper panels), diabetic rats exhibited reduced insulin expression and secretion in the DG region of the HPC (Figure 4A, lower panels), and detected signals were almost completely colocalized with EAAT3 proteins, which were highly upregulated in the diabetic HPC. Magnified confocal images of IHC sections indicate that EAAT3 and insulin colocalized specifically in the diabetic HPC (Figure 4B, right panels) and that, although HPC cells derived from WT rats expressed higher levels of insulin, this upregulated insulin did not colocalize with EAAT3 (Figure 4B, left panels).

In IHC analysis of OB tissues from both WT and diabetic rats, we observed similar alterations in the expression of EAAT3 protein. In the WT OB, insulin was highly expressed, whereas EAAT3 was expressed at low levels (Figure 5A, upper panels, and Figure 5B, left panels). In contrast, the OB from diabetic rats exhibited upregulation of EAAT3 protein, and EAAT-positive signals were partly colocalized with those of insulin (Figure 5A, lower panels, and Figure 5B, right panels). Diabetes-specific colocalization of EAAT3 and insulin was further examined by immunoprecipitation analysis (IP). Pulled-down fractions of HPC and OB lysates with monoclonal anti-insulin antibodies demonstrated that EAAT3 interacted with insulin specifically in diabetic samples (Figures 4C and 5C). Similarly, pulled-down fractions of HPC and OB lysates with anti-EAAT3 antibodies demonstrated that insulin interacted with EAAT3 in diabetic samples (Figures 4C and 5C). Our IP analysis suggested that EAAT3 protein could bind to and capture insulin in the diabetic state. This association between EAAT3 and insulin was specifically observed in the HPC and OB from diabetic rats when EAAT3 expression was highly upregulated (Figures 4C and 5C).

Functions of glutamate transporters include regulation of excitatory neurotransmission, maintenance of low ambient extracellular glutamate concentrations to protect against neurotoxicity, and providing glutamate for metabolism through the glutamate-glutamine cycle. Hyperactivity of glutamate transporters has been implicated in the pathophysiology of schizophrenia and other mental illnesses [45]. In the pancreatic islets of Langerhans, glutamate is proposed to act as an intracellular messenger, regulating insulin secretion from β cells. EAAT regulates the pH and membrane potential of the granules and thereby regulates insulin secretion in pancreatic β cells. Increased EAAT expression during the progression of diabetes in adult OB and HPC neurons (Figures 3, 4 and 5) may cause not only unbalanced glutamate-mediated transmission in the CNS but also the dysregulation of insulin secretion from neurons [29]. Our data may therefore be important for understanding the novel functions of identified genes associated with diabetes-related neurodegenerative disorders in the CNS in future studies.

## Conclusions

Adult NSCs extracted from the OB and HPC of diabetic rats at early phases of diabetes progression can be expanded in *in vitro* cultures. Thus, because OB NSCs reacted similar to HPC NSCs in terms of differentiation potential and gene expression, these cells represent a useful tool to investigate the neurogenic functions of the CNS and to develop potential drugs for the treatment of clinical disorders. In particular, *Nrxn1* and *Scn1b* transcripts were downregulated, whereas EAAT3 protein and mRNA were upregulated in both the HPC and OB of diabetic rats. These alterations suggest that the HPC and OB may exert similar influences on the progression of diabetes.

In future studies, elucidating the pathology of diabetes-mediated neurodegenerative disorders, neurologic diseases, and mental illnesses, the risks of which increase as the pathology of diabetes progresses, or searching for new therapeutic reagents for the treatment of diabetes itself and developing novel treatment techniques may all be facilitated through the use of NSCs derived from the OB.

## Abbreviations

BYSL: Bystin-like; Cask: Calcium/calmodulin-dependent serine protein kinase; CNS: Central nervous system; DB: Diabetes; DG: Dentate gyrus; Dvl: Disheveled; EAAT: Excitatory amino acid transporter; FGF-2: Fibroblast growth factor 2; Fzd: Frizzled; GFAP: Glial fibrillary acidic protein; HPC: Hippocampus; LRP: Low-density lipoprotein receptor-related protein; Nid2: Nidogen 2; NSC: Neural stem cell; Nrxn1: Neurexin 1; Nupr1: Nuclear protein 1; OB: Olfactory bulb; Scn1b: Sodium channel, voltage-gated, type I, β subunit; Scn4b: Sodium channel, voltage-gated, type IV, β subunit; Snai1: Snail homolog 1, zinc-finger protein; SSH: Sonic hedgehog; STZ: Streptozotocin; SVZ: Subventricular zone; SYN1: Synapsin 1; TUBB3: β-tubulin III; XT: Wild type.

## Competing interests

The authors declare that they have no competing interests.

## Authors’ contributions

TK was involved in the designing of research, data analysis, and manuscript writing. RH, MM, SF, and KT performed research and contributed to acquisition of data. MA designed research and supported the research and the resources. All authors read and approved the manuscript for publication.
